# A Manual of Venereal Diseases

**Published:** 1892-05

**Authors:** 


A Manual of Venereal Diseases: Being an Epitome of the Most Approved Treatment. By Everett M. Culver, A. M., M. D., Pathologist and Assistant Surgeon Manhattan Hospital of New York City; Member of the American Association of Andrology and Syphilology, and late of the Department of Venereal Diseases of the Vanderbilt Clinic; and James R. Hayden, M. D., Chief of Clinic Venereal Department of Vanderbilt Clinic, College of Physicians and Surgeons, New York. With illustrations. Philadelphia: Lea Brothers & Co. 1891.

This book is a practical treatise, presenting in a condensed form the essential features of our present knowledge of the three venereal diseases, syphilis, chancroid, and gonorrhea. For a long period of time, even up to within a very recent date, the ravages of syphilis were considered to be of much more importance and much more destructive to the human race than those of gonorrhea; but since the studies of Neisser, and the promulgation of the doctrines of Noeggerath, gonorrhea and syphilis have changed places with reference to their importance, prevention, and dangers. An uncured gonorrhea is capable of inflicting more permanent mischief upon the innocent members of society than almost any other disease that can be named.

It is a great question whether there should not be some established rule by which innocent women can be prevented from marrying with men who are already contaminated, and will surely infect a previously healthy and innocent wife. This is a matter of so much importance that every practitioner, especially in the smaller cities and towns, and even in the rural districts, should familiarize himself with the methods of diagnosis, prevention and treatment of the three maladies that are so ably handled by the authors of this book. We have examined this work carefully, and have come to the conclusion that it is the most concise, direct, and able treatise that has appeared on the subject of venereal diseases for the general practitioner to adopt as a guide. The specialist, of course, must possess himself of exhaustive treatises, and familiarize himself with the technique and minutia of his craft, but the general practitioner needs a few simple, concise, and clearly presented laws, in the execution of which he cannot fail to either cure or prevent the ravages of the maladies in question and the direful results which their propagation entails.

